# Characterization of the Flux System: Lithium-Aluminum Silicate (Li)–Alkali Feldspars (Na,K); Magnesium (Mg) and Calcium (Ca)–Silicates

**DOI:** 10.3390/ma14237386

**Published:** 2021-12-02

**Authors:** Agata Stempkowska

**Affiliations:** Department of Environmental Engineering, Faculty of Civil Engineering and Resource Management, AGH University of Science and Technology, Mickiewicza 30 Av., 30-059 Cracow, Poland; stemp@agh.edu.pl

**Keywords:** flux system, mineral eutectic, thermal properties

## Abstract

In this paper, the system of natural mineral alkali fluxes used in typical mineral industry technologies was analyzed. The main objective was to lower the melting temperature of the flux systems. The research has shown that the best melting parameters in the Ca–Mg– (Li,Na,K) system were characterized by the composition: A-eutectic 20% and wollastonite 80%, and it was reached at temperature 1140 °C; in addition, this set had the widest melting interval. Selected thermal parameters of mineral flux systems were also calculated. The technological properties of mineral composites such as shrinkage and brightness were also analyzed.

## 1. Introduction

Feldspar raw materials play an important role in the modern mineral industry. They also contribute significantly to lowering the sintering temperature of various mineral materials. Low-melting sodium-potassium aluminosilicates dissolve large amounts of quartz and clay substances, causing densification of the material and creating conditions for recrystallization. Feldspar raw materials are rich in alkalis (K_2_O + Na_2_O), which are mainly bound in the form of aluminosilicates, i.e., potassium feldspars (orthoclase, microcline, sanidine, adular) and sodium-calcium feldspars. Albite was used as the sodium substrate of the study. Stochiometric albite contains 11.8%wt Na_2_O, 19.4%wt A1_2_O_3_ and 68.8%wt SiO_2_. Its melting point is 1120–1200 °C, and its density is 2.62 g/cm^3^. The characteristic of albite is lower melt viscosity compared to other feldspars. In general, it is a better flux than potassium feldspar, because it melts at a lower temperature, but causes a greater deformation in the material due to congruent melting [1,2]. The potassium substrate was orthoclase, which stoichiometrically contains K_2_O 16.9%wt, Al_2_O_3_ 18.3%wt and SiO_2_ 64.8%wt. The average density of orthoclase is 2.59 g/cm^3^. During the sintering of materials, the silicate alloy formed at the expense of feldspar interacts with the solid phase and partially dissolves it. This phenomenon begins at a temperature of about 1150 °C. Silica alloy leads to the expected densification and lower porosity of the material [3,4]. The authors of previous studies found that in aluminosilicate systems, the Na_2_O/K_2_O ratio has a significant influence on the sintering temperature. The most favorable is when this ratio is 2, then the firing temperature decreases by about 25 °C. It is worth noting that the smaller the value of the Na_2_O/K_2_O ratio, the smaller the deformation of plastic. This effect decreases with the fineness of the system [5,6,7].

In the author’s previous research, a basic flux system based on sodium-potassium lithium aluminosilicates was analyzed, using naturally occurring raw materials such as spodumene, albite, and orthoclase, an attempt was made to obtain the eutectic with the lowest melting point [8]. Lithium was introduced into the masses in the form of spodumene, which occurs in relatively pure form in nature (Greenbushes, Australia) [9,10]. The use of lithium in the form of carbonate for high-temperature processes (sintering) can cause a problem with outgassing of the resulting CO_2_ [11,12,13]. Spodumene is one of three natural varieties of lithium aluminum silicate, the others being petalite and eucryptite. Spodumene melts at a temperature of about 1420 °C. However, in combination with quartz, feldspars, and mica, it forms low-temperature eutectics [8,14,15].

The proportion of CaO and MgO oxides also has a significant effect. In the case of CaO, the sintering temperature decreases further (by about 10 °C) in contrast to MgO. It was also found that CaO and MgO oxides do not influence the temperature deformation of the polymer [16]. Talc is often used as a magnesium substrate. Talc is a magnesium hydrosilicate with the theoretical formula 3MgO–4SiO_2_–H_2_O, which corresponds to 63.35 wt% SiO_2_, 31.90 wt% MgO and 4.75 wt% chemically bound water. On the other hand, other minerals such as chlorite, mica, feldspars, rutile, pyrite, carbonates, pyrite, magnetite and hematite can be associated with talc, and these admixtures can significantly affect its thermal properties [17]. The influence of magnesium is interesting when using a silicate such as talc, as materials of increased densification and mechanical properties are obtained. However, studies on the effect of a heating cycle (heating rate and heating time), purity of talc materials (including calcination) and related thermal properties are very important [18,19]. In glass-ceramic materials using talc, mineral bridges and nanocrystallites have been found to cause crack deflection, which strengthens the grain boundary and improves the mechanical properties of the ceramics [20,21]. Calcium can be introduced into the system in the form of wollastonite. Wollastonite is a calcium silicate mineral, either natural or synthetic. Commercial wollastonite begins to melt at about 1450 °C and cannot be considered a “flux” such as alkali feldspar [22,23,24]. In view of this, the purpose of this study emerged, which was to investigate the mechanism of action of wollastonite as a ceramic flux. The application of wollastonite in mineral systems was investigated by analyzing its reactivity with other materials such as spodumene, talc and feldspars. Ca–Mg silicates, especially wollastonite, are of increasing interest in the field of bioceramics, mainly due to their bioactivity and biocompatibility. The obtained whiteness of the mineral sinters is also an important feature [25,26,27,28,29,30,31]. The main objective of this study was to determine the temperature pattern of phase transformation-eutectics and to calculate and visualize the thermal parameters of the eutectic transformation.

## 2. Materials and Methods

**Figure 1:**
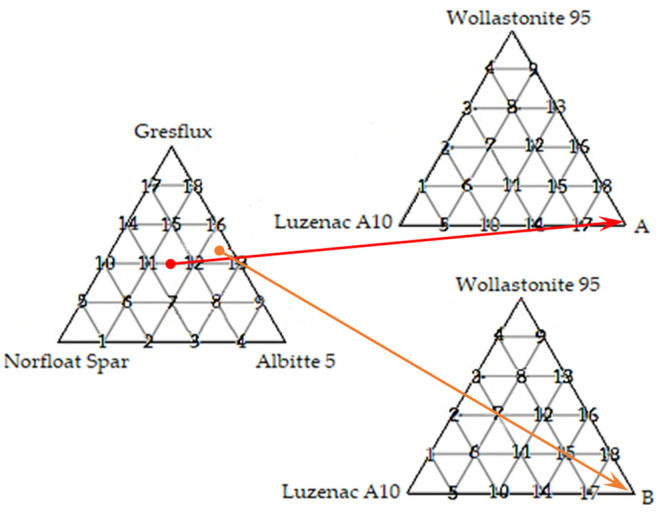
Graphical presentation of the arrangement of mineral fluxes.

In this study, the effect of selected Ca and Mg alkali earth silicates on the melting point of the spodumene/Na-feldspar/K-feldspar system was analyzed using commercial raw materials: Concentrate, Albitte 5, Norfloat Spar, Wollastonite 95 and Luzenac A10H supplied by Otavi Minerals (Neuss, Germany). Chemical analyses of the studied raw materials in conversion to oxides are presented in Table 1. The existence of two eutectic points in the Li–Na–K system gave rise to two new flux compositions. The connection between the previous studies is shown graphically in Figure 1. [8] A-eutectic had the following composition: 30% potassium feldspar, 30% sodium feldspar, 40% spodumene, and B-eutectic: 5% potassium feldspar, 45% sodium feldspar, 50% spodumene (respectively Albitte 5, Norfloat Spar, Gresflux). Two sets of 18 compositions differing by 20% by weight were prepared. The arrangement of the samples in the triangle of compositions is also shown in Figure 1.

### 2.1. High-Temperature Microscopy

On individual sets of specimens, measurements were performed using a high-temperature microscope (Hesse-Instruments, Osterode am Harz, Germany) with the following assumptions; average temperature increments of 10 °C/min in the temperature range from 80 to 1500 °C. On the basis of continuous observation of the sample and recording changes in its dimensions as a function of temperature, the so-called characteristic temperatures were determined:

- Shrinkage temperature T_g_ (sintering),
- Softening temperature T_a_ (corner rounding-end of sintering),
- Melting temperature T_b_ (hemisphere effect-melting),
- Spreading temperature T_c_ (sample base >200% or 1/3 height),

and also:

- Sintering interval (corner rounding temperature T_a_—sintering temperature T_g_),
- Melting interval (hemisphere temperature T_b_—corner rounding temperature T_a_),

Investigations carried out in the high-temperature microscope belong to the standard investigations of thermal properties of materials. They allow not only the determination of characteristic temperatures but also the determination of decomposition temperatures, sublimation or phase transition temperatures, etc. [32,33]. Data visualization was performed with the Surfer 19 program from Golden Software. The test results presented are averages of three measurements.

### 2.2. Selected Thermal Parameters of Mineral Flux Systems

The energy transformation of a body during its heating or given off during its cooling is the product of the mass of the material *m* and the temperature difference of that material Δ*T* before and after the heat transformation. The principle of thermal transformation capacity Δ*Q* can be written in the form:

Δ*Q* = *c_v_*·*m*·Δ*T* (J) (1)

The heat capacity *c_v_* that accrues per unit mass of a substance is called the specific heat (J/kg·K). The specific heat is an additive quantity, i.e., each factor present in a system contributes to the total heat of that system. [34,35,36]. Another quantity that characterizes materials in terms of their thermal properties is the volumetric heat capacity. Its value, *b*, is calculated as the product of the specific heat *c_v_* and the density *ρ* of the material from which the material is made:

*b = c_v_·*ρ (J/(m^3^K)) (2)

Volumetric heat capacity is a measure of the amount of energy that 1 m^3^ of a given material will absorb while heating or lose while cooling, changing its temperature by one degree. The highest heat capacity is characteristic for materials of the highest density. The eutectic transformation during heating requires a certain heat power *P*, which is determined by the relation in which Δ*Q* is the emitted energy (1), and *t* is the heating time [37]:

*P* = ∆*Q*/t (J/s) (3)

### 2.3. Selected Physical Parameters

For visual and other physicochemical properties studies, 30 mm diameter discs were pressed from each set. For each of the 18 sets, 2 discs were pressed for both the first and second triangular compositions. The samples were molded in special steel molds at a pressure of 35 MPa and then were fired in a Nabertherm LH 30/14 laboratory chamber furnace (Frankfurt, Germany).

Linear shrinkage was determined from the changes in the diameter of discs fired at different temperatures. Measurements were made by measuring the diameter three times in different directions and using Formula (4):

(4) $$S_{w}=\frac{M-S}{M}\cdot 100$$

where:

S_w_—linear shrinkage (%),

M—diameter of the pastille after forming and drying (mm),

S—diameter of the pastille after firing (mm).

Luminance (whiteness) was measured with a Zeiss Jena leucometer (Warsaw, Poland), and this parameter depends mainly on the purity (content of coloring compounds) of the starting components. The *L a b* system is currently the most popular way to describe color and is the basis of modern color diagnostic systems, allowing for additional independence in color identification from the class of instrument (e.g., camera or spectrometer). This system extends between opposing colors, forming the following three dimensions: the *L* dimension denoting brightness, and the *a* and *b* dimensions [38,39].

*L*—the brightness of the color within the values from 0 to 100, 

*a*—the percentage of green or red in the color under consideration, while the green hue is negative and the red hue is positive,

*b*—the proportion of blue or yellow in the color under analysis, whereby the blue hue has a negative value, and the yellow hue has a positive value.

## 3. Results and Discussion

### 3.1. Visualization of Thermal Parameters Obtained from a High-Temperature Microscope of a Three-Component System

#### 3.1.1. Characteristic Temperatures of the System: A-Eutectic–Talc–Wollastonite and B-Eutectic–Talc–Wollastonite

**Figure 2:**
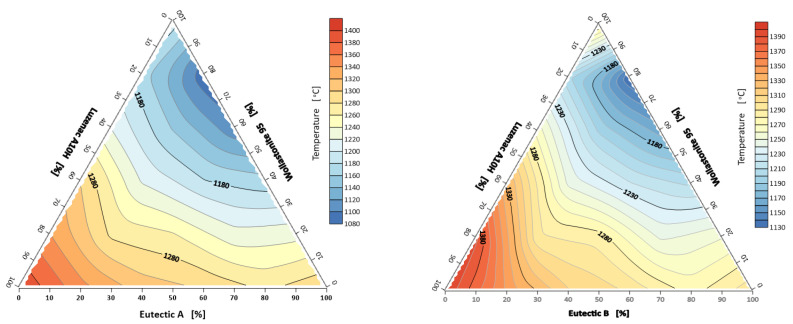
Corner rounding temperatures: A/B-eutectic–Luzenac A10H–Wollastonite 95.

**Figure 3:**
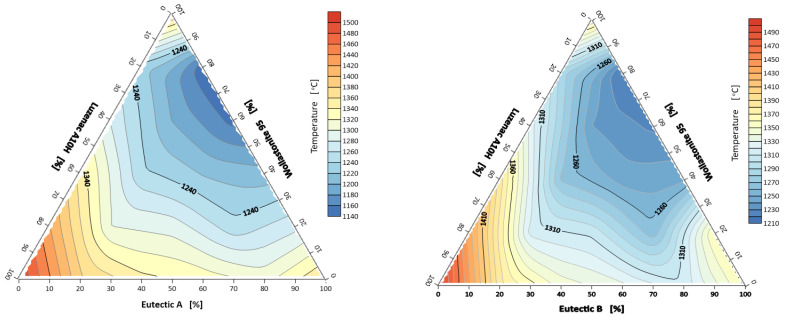
Melting points (hemisphere) of the system: A/B-eutectic–Luzenac A10H–Wollastonite 95.

As can be seen from the conducted tests for the first system (A-eutectic–talc–wollastonite) the lowest sintering temperature (rounding of corners) was registered for the sample with the composition: 80% A-eutectic, 20% Wollastonite 95, and it was 1062 °C, while the highest temperature of 1399 °C was recorded for the sample containing 100% Luzenac A10H (Figure 2). For the second flux system (B-eutectic–talc-wollastonite), as before, the lowest sintering temperature of 1130 °C was observed for the composition of 80% B-eutectic and 20% Wollastonite 95 (Figure 2.). The results indicate an adverse effect of the increased amount of talc in the studied flux system. In both the first and second sets, talc significantly increases the sintering temperature. The results are different with wollastonite, whose addition improves the sintering of the system. The data analysis shows that talc has the highest melting point (hemisphere) of the minerals studied. Thus, it is an undesirable additive because it does not produce an increased amount of liquid phase. However, it can be observed (Figure 3) that a suitable combination of the raw materials spodumene, potassium and sodium feldspar and wollastonite significantly reduces the melting point. The melting point of A-eutectic 80% and 20% wollastonite was about 1140 °C, while that of 80% B-eutectic and 20% wollastonite was about 1210 °C, respectively, and the melting point of the studied wollastonite (starting raw material) is 1361 °C. Previous studies by the author on the selection of the optimal composition of A-eutectic fluxes (40% spodumene, 30% potassium feldspar, 30% sodium feldspar) showed that the lowest melting point was 1263 °C [8] (Table 1).

**Figure 4:**
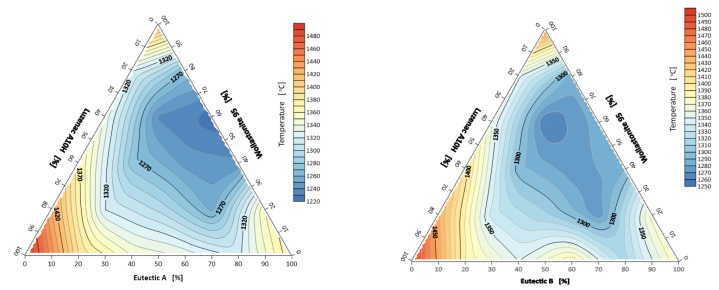
System spreading (flow) temperature: A/B-eutectic–Luzenac A10H–Wollastonite 95.

The first of the composition triangles: A-eutectic–talc–wollastonite, is characterized by the lowest spreading temperature of 1223 °C at the point corresponding to the 60/0/40% composition, respectively. The highest melting temperature, i.e., 1429 °C, was obtained for the composition of 20% A-eutectic and 80% talc (Figure 4). In the case of the system B-eutectic–talc–wollastonite, the lowest and the highest flow temperatures were recorded at different points than in the previous case. The lowest spreading temperature of 1227 °C was recorded at the point corresponding to the composition of 60% B-eutectic, 20% talc and 20% wollastonite and the highest temperature of 1460 °C was observed at the point of 100% B-eutectic. The measurements showed that also in this case, as the amount of talc in the composition increases, the melting temperature increases (Figure 4).

The study showed that the melting temperature of the different sets with A and B eutectic measured as the point of hemisphere formation between the two composition triangles did not differ significantly.

#### 3.1.2. Melting and Flow Intervals of the System A-Eutectic–Talc–Wollastonite and B-Eutectic–Talc–Wollastonite

**Figure 5:**
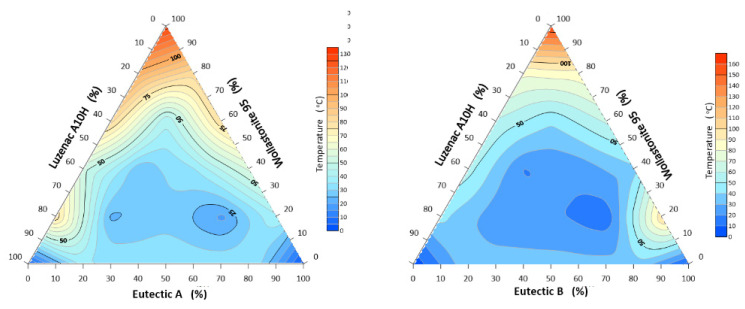
Sintering interval of the system: A/B-eutectic–Luzenac A10H–Wollastonite 95.

**Figure 6:**
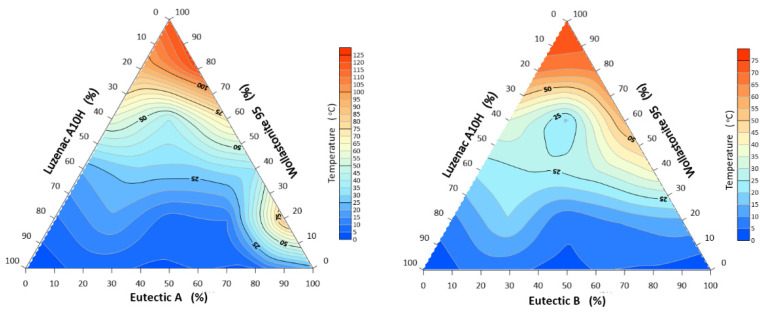
System melting interval: A/B-eutectic–Luzenac A10H–Wollastonite 95.

The sintering interval was calculated as the difference between the characteristic corner rounding temperature T_a_ and the maximum shrinkage temperature T_g_. This temperature range is not always determined in flux systems but gives an idea of the possible deformation of the plastic. For the triangle of compositions with flux A, the narrowest sintering interval 18.5 °C was recorded for the composition 20% A-eutectic/60% Wollastonite 95/20% Luzenac A10H, and the widest interval occurred for the composition 100% A-eutectic and was 115 °C (Figure 5). The second composition system; B-eutectic–Luzenac A10H–Wollastonite 95 has a wide melting interval of about 100 °C at the 100% B-eutectic point. The narrowest sintering interval of 14 °C occurred for a composition of 20% B-eutectic/60% Luzenac A10H/20% Wollastonite 95. In the mineral industry, it is important not only to select effective fluxes that lower the melting point but also to extend its flow. The results are shown below in Figure 6. For the system A-eutectic–talc–wollastonite, the narrowest melt interval of about 3 °C occurred at the point with a composition of 20% wollastonite and 80% talc. The widest interval of 123 °C was recorded for the composition of 80% A-eutectic and 20% wollastonite.

For the system B-eutectic–talc–wollastonite, the narrowest interval of 4 °C was recorded for the composition of 80% wollastonite and 20% talc. For the 100% B composition point, the widest melting interval of 75 °C occurred.

### 3.2. Thermal Parameters of the Eutectic System

Based on the percentage of individual oxides contained in the raw materials (Table 1) and their tabulated ʼ values (Table 2), the average specific heat of the materials studied was calculated (Table 3). Specific heat is a quantity particularly sensitive to phase transformations. It carries valuable information about the nature, latent heat or critical exponent of the transformation. The average specific heat closely depends on the chemical composition of the raw material. Table 3 also shows the average density of the studied systems necessary for further calculations.

The materials with the highest specific density are characterized by the highest thermal accumulation capacity *b*. Analyzing the values of this parameter in Table 4, it was found that the flux system is characterized by similar values to natural rocks. For example, natural rock formations have lower volumetric heat capacity than metals, e.g., granite-about 1.8 MJ/(m^3^ K) gabbro about 2.2 MJ/(m^3^ K), granodiorite about 2.3 MJ/(m^3^ K). Even lower volumetric heat capacity is characteristic for brick and sand—about 1.2 MJ/(m^3^ K) [40,41,42]. The transformation energy of the ternary mixture depends on the mass m, the average specific heat c_v_ and the temperature difference during thermal exposure. The mass of the samples remained constant. The temperature difference is the beginning of the heating of the system and reaching the melting temperature T_b_, i.e., the phase transformation. From a technological point of view, the lowest energy value is the most desirable—a mineral melt is obtained with the minimum possible heat supply. Table 4 shows the values of the thermal transformation energy of the system ∆*Q*, it can be noted that the lowest value (808 kJ) was observed for the system containing 40% eutectic A and 60% talc. The highest value (1018 kJ) occurred at the same point with a composition of 80% wollastonite and 20 eutectic A/B in both cases.

**Figure 7:**
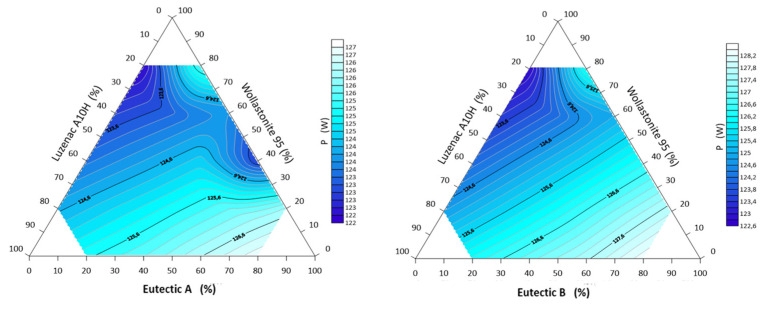
Thermal power *P* of three-component mineral system Eutectic A/B–Luzenac A10H–Wollastonite 95.

Figure 7 shows the heatpower map of the ternary system Eutectic A/B–Luzenac A10H–Wollastonite 95. For the system containing eutectic A, two fields of lowest heatpower values can be observed for flux compositions of 20% talc and 80% wollastonite, and 40% wollastonite and 60% eutectic A. For the second system, the heatpower generally decreases with wollastonite content.

### 3.3. Characteristics of Other Parameters of Alloys of the System: A-Eutectic–Talc–Wollastonite and B-Eutectic–Talc–Wollastonite

#### 3.3.1. Linear Shrinkage of Alloys of the System: A/B-Eutectic–Talk–Wollastonite

**Figure 8:**
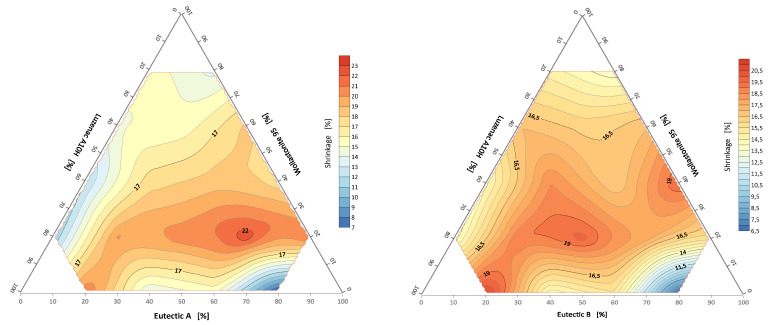
Shrinkage at corner rounding temperature of system: A/B-eutectic–Luzenac A10H–Wollastonite 95.

The sintering shrinkage was calculated as the percentage change in the width and height dimensions of the specimens at the corner rounding temperature T_a_ from the starting dimensions. The shrinkage map is shown in Figure 8. For the set with eutectic A, the field of highest shrinkage was observed for the set with a composition of talc 20%, wollastonite 20% and eutectic A 60%. For the second set, the fields of highest shrinkage are arranged linearly for three sets with compositions of 40% wollastonite–60% eutectic B, 40% talc–20% wollastonite–40% talc, 80% talc–20% eutectic B.

**Figure 9:**
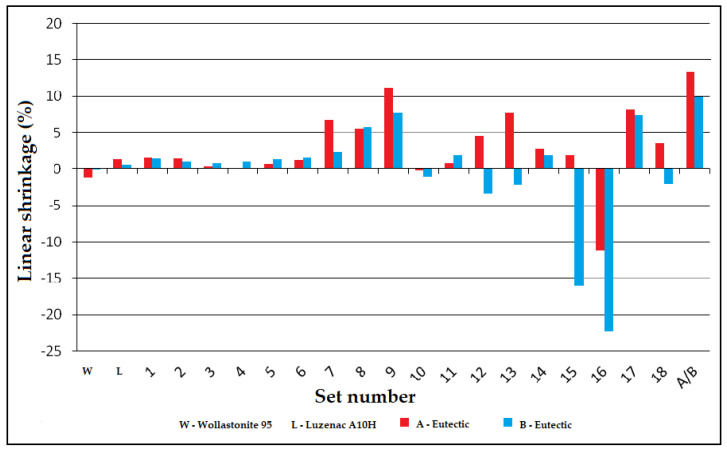
Comparison of the linear shrinkage of the A/B eutectic–wollastonite–talc.

Shrinkage tests were repeated on samples formed by pressing. The lozenges of the triangular composition A-eutectic–talc–wollastonite were fired at 1145 °C, and the triangular composition of B-eutectic–talc–wollastonite at 1218 °C. These temperatures are the lowest determined hemisphere temperatures (Figure 3). The highest linear shrinkage was observed for the composition containing a 100% A-eutectic set and was 13.5%, and for the set containing 100% B-eutectic this value was 9.9% (Figure 9). The second highest was set 9 for A-eutectic (60% A-eutectic and 40% wollastonite) in which the shrinkage was 11.3% and sets 9 and 13 with a shrinkage of 7.5%, and in sets 15 and 16, swelling of the samples was observed.

#### 3.3.2. Whiteness of Alloys: A/B-Eutectic–Talc–Wollastonite

**Figure 10:**
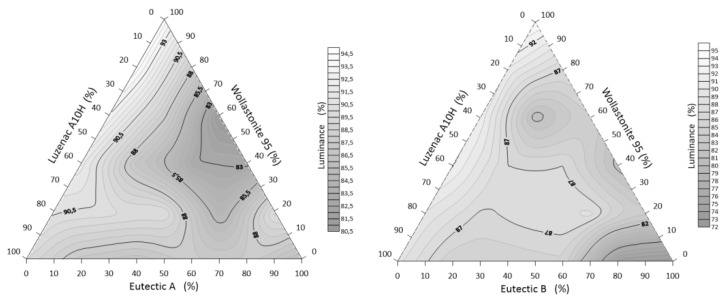
Whiteness of the system A/B-eutectic–talc–wollastonite.

For the selected starting components, the luminance (a measure of brightness measured in the lab system) of the alloys decreases as the melting temperatures decrease. The relationship between the value of the whiteness parameter L and the individual compositions is shown graphically (Figure 10).

From the obtained data, it can be read (Table 5) that the highest value of the color parameter L = 94.49% was registered for the sample No. 1 with the composition of 80% Wollastonite 95 and 20% Luzenac A10H (the sample does not contain a Li–Na–K mixture in its composition), and the lowest value of 72.45% for the composition of 18 B-eutectic. The whiteness depends on the degree of sintering of the sets and their compositions. The measurements showed the highest whiteness for the sets containing increased amounts of wollastonite. Furthermore, talc clearly improves the whiteness. In general, the lower the degree of sintering, the higher the whiteness.

## 4. Conclusions

- The lowest melting point was observed for the composition of A-eutectic 20% and wollastonite 80% and it was 1140 °C. Similarly, for the system with B-eutectic, the lowest melting point of 1210 °C was recorded for the point with the same composition.
- An increase in the talc content of the flux system increases the characteristic temperatures. Wollastonite improves the sintering of the system.
- From the technological point of view, the flow interval is important, and the most favorable was again the point A-eutectic 20% and wollastonite 80% (set number 9) with the interval of 123 °C.
- The lowest transformation energy of the system Δ*Q* 808kJ was recorded for the point 40% A-eutectic and 60% wollastonite, at the same time the sets A/B-eutectic 20% and wollastonite 80% in which the eutectic point was recorded to have the highest transformation energy 1018 kJ.
- Maximum shrinkage occurs at the same points for the A/B-eutectic –wollastonite–talc system (set 9).
- The most promising from the point of view of industrial implementation seems to be set 9, i.e., 80% wollastonite with eutectic A. This set has the lowest melting point and the highest density.
- Currently, technological (strength, water absorbability, density) and microstructural studies are carried out on the obtained lithium aluminum silicate (Li)–alkali feldspars (Na,K)–magnesium (Mg) and calcium (Ca) silicates.

## Figures and Tables

**Table 1:** Chemical composition of raw materials applied.

Raw Material	Wollastonite 95	Luzenac A10H	Eutectic A	Eutectic B
	Chemical Composition (%wt.)			
SiO_2_	50.98	61.0	67.1	67.49
Al_2_O_3_	0.41	0.3	19.09	20.19
**CaO**	**45.54**	**0.4**	**0.49**	**0.50**
**MgO**	**1.20**	**32.0**	**0.5**	**0.69**
TiO_2_	-	-	0.11	0.16
Fe_2_O_3_	0.29	0.2	0.44	0.53
MnO	-	-	0.08	0.1
P_2_O_5_	0.11	-	0.18	0.21
**Na_2_O**	**0.19**	**0.1**	**4.12**	**4.92**
**K_2_O**	**0.12**	**-**	**4.06**	**1.19**
**Li_2_O**	**-**	**-**	**2.44**	**3.05**
LOI	1.15	6.0	1.49	1.02
Metlig Point	1450 °C	1500 °C	1263 °C	1376 °C
Dominant Mineral	wollastonite (95%)	talc (95%)	-	-

Bold is the most important oxides for this work.

**Table 2:** Specific heat of oxides in the raw materials.

Oxide	c_v_ (J/kg·K)
SiO_2_	742
Al_2_O_3_	775
Fe_2_O_3_	655
MgO	924
CaO	750
Na_2_O	1115
K_2_O	764
Li_2_O	1811

**Table 3:** Calculated average specific heat c_v_ and density ρ of used fluxes.

Specific Heat c_v_	Wollastonite 95	Luzenac A10H	Eutectic A	Eutectic B
(J/kg K)	742.69	765.06	778.26	790.00
(g/cm^3^)	2.98	2.71	2.79	2.87

**Table 4:** Volume heat capacity *b*, time *t* of eutectic formation, and transformation energy *∆Q* of the investigated sets.

Set Number	Eutectic A–Luzenac A10H–Wollastonite 95			Eutetic B–Luzenac A10H–Wollastonite 95		
	B MJ/(m^3^K)	t sec	∆Q kJ	b M J/(m^3^K)	t sec	∆Q kJ
1	2.098	6774	843	2.098	7656	955
2	2.127	7164	888	2.127	7788	966
3	2.155	7794	961	2.155	7872	971
4	2.182	8046	987	2.182	8088	992
5	2.090	6372	799	2.110	7398	934
6	2.119	6948	868	2.137	7368	924
7	2.148	7308	909	2.166	7584	946
8	2.176	7656	947	2.194	7656	950
9	2.256	8082	1018	2.275	8052	1018
10	2.112	6414	808	2.148	6900	876
11	2.141	7122	893	2.177	7092	895
12	2.170	7572	945	2.206	7308	917
13	2.200	7788	967	2.234	7794	974
14	2.133	6930	877	2.186	6420	819
15	2.163	7368	928	2.216	6948	882
16	2.156	7566	932	2.245	7164	905
17	2.155	7398	940	2.226	6372	818
18	2.185	7482	946	2.256	6780	866

**Table 5:** Values of color components (%) in the system L a b of the investigated sets.

Set Number	Eutectic A–Luzenac A10H–Wollastonite 95			Eutectic B–Luzenac A10H–Wollastonite 95		
	L (%)	a (%)	b (%)	L (%)	a (%)	b (%)
1	**94.49**	0.69	7.34	91.12	0.79	13.49
2	93.09	1.69	8.95	92.01	0.89	11.50
3	91.78	2.60	10.40	**92.23**	1.53	13.22
4	90.63	3.24	11.83	89.94	2.39	14.91
5	88.30	−0.82	10.57	87.08	−0.96	9.15
6	88.27	−0.24	11.51	81.25	−0.14	16.20
7	86.85	−0.49	12.58	87.01	0.62	14.52
8	90.26	0.79	11.03	86.80	0.75	16.20
9	87.15	0.80	9.44	85.07	1.79	12.07
10	**80.58**	−0.60	1.71	84.94	−1.46	4.00
11	83.19	−0.75	9.59	87.00	−0.78	13.46
12	90.00	−0.03	10.63	87.79	−0.08	11.10
13	86.50	1.25	10.10	85.15	1.40	11.62
14	82.80	−1.53	2.26	81.30	−2.70	1.81
15	84.68	−0.51	8.37	88.21	−0.78	7.10
16	87.70	0.65	9.07	85.71	0.15	8.45
17	90.38	−0.33	6.81	84.18	−0.96	4.79
18	87.70	−2.55	1.12	**72.45**	−1.93	1.67
Wollastonit	94.49	0.31	6.09	94.49	0.31	6.09
Talk	89.53	3.85	12.33	89.53	3.85	12.33
A/B	85.61	−0.03	2.02	71.41	−0.16	1.64

Bold: highlight the most important results.

## Data Availability

Data available on request due to restrictions. The data presented in this study are available on request from the corresponding author. The data are not publicly available due to their potential use and implementation on an industrial scale.
